# Exploring Factors Associated with Patients Who Prefer Clinician-Sampling to HPV Self-Sampling: A Study Conducted in a Low-Resource Setting

**DOI:** 10.3390/ijerph19010054

**Published:** 2021-12-22

**Authors:** Jessica Sormani, Bruno Kenfack, Ania Wisniak, Alida Moukam Datchoua, Sophie Lemoupa Makajio, Nicole C. Schmidt, Pierre Vassilakos, Patrick Petignat

**Affiliations:** 1 Gynaecology Division, Department of Paediatrics, Gynaecology and Obstetrics, University Hospitals of Geneva, 1205 Geneva, Switzerland; ania.wisniak@hcuge.ch (A.W.); Sophie.Lemoupa@hcuge.ch (S.L.M.); Nicole.schmidt@ksh-m.de (N.C.S.); pierrevassilakos@bluewin.ch (P.V.); Patrick.petignat@hcuge.ch (P.P.); 2 School of Health Sciences, HES-SO University of Applied Sciences and Arts Western Switzerland, 1227 Geneva, Switzerland; 3 Department of Obstetrics and Gynecology, Faculty of Medicine and Pharmaceutical Sciences, University of Dschang, Dschang, Cameroon; brunokenfack@gmail.com; 4 Department of Gynaecology and Obstetrics, District Hospital of Dschang, Dschang, Cameroon; moukamalida@gmail.com; 5 Faculty of Medicine, Institute of Global Health, University of Geneva, 1205 Geneva, Switzerland; 6 Faculty of Social Science, Catholic University of Applied Science, 55122 Mainz, Germany; 7 Geneva Foundation for Medical Education and Research, 1202 Geneva, Switzerland

**Keywords:** cervical cancer screening, HPV self-sampling, sub-Saharan Africa, preference

## Abstract

Human papillomavirus (HPV) self-sampling (Self-HPV) is a promising strategy to improve cervical cancer screening coverage in low-income countries. However, issues associated with women who prefer conventional HPV clinical-sampling over HPV self-sampling may affect screening participation. To address this issue, our study assessed factors associated with women’s preferences related to Self-HPV. This study was embedded in a large clinical trial recruiting women aged 30–49 years in a primary HPV-based study termed “3T-Approach” (for Test-Triage-Treatment), launched in 2018 at Dschang District Hospital, West Cameroon. Participants were invited to perform a Self-HPV. After the sampling and before receiving the results, participants completed a questionnaire about cervical cancer screening and their preferences and perceptions around Self-HPV. The median age of the 2201 participants was 40.6 (IQR 35–45) years. Most (1693 (76.9%)) preferred HPV self-sampling or had no preference for either method, and 508 (23.1%) preferred clinician-sampling. Factors associated with an increased likelihood of reporting a clinician-sampling preference were tertiary educational level (29.4% CI: 25.6–33.6 vs. 14.4% CI: 12.8–16.1) and being an employee with higher grade professional or managerial occupations (5.5% CI: 3.8–7.9 vs. 2.7% CI: 2.0–3.5). The main reported reason for women preferring clinician-sampling was a lack of “self-expertise”. Most women (>99%) would agree to repeat HPV self-sampling and would recommend it to their relatives. HPV self-sampling in the cultural context of central Africa was well accepted by participants, but some participants would prefer to undergo clinician sampling. Health systems should support well-educated women to increase self-confidence in using HPV self-sampling.

## 1. Introduction

Cervical cancer (CC) is the second most common cancer in Cameroon, with 2356 cases and 1787 deaths recorded in 2020 [1]. In sub-Saharan Africa, CC is the leading cause of cancer deaths in women, despite being a highly preventable disease [2]. The widespread understanding that almost all cervical cancer cases occur in women who were previously infected with human papillomavirus (HPV) has resulted in the development of HPV tests and primary HPV-based cervical cancer screening. Furthermore, HPV testing is performed on vaginal smear samples, which has the advantage of allowing collection by patients themselves (Self-HPV).

The World Health Organization (WHO) launched a global initiative in 2020 to eliminate cervical cancer and includes Self-HPV as a part of cervical cancer screening programs [3,4]. Systematic reviews support the finding that Self-HPV has similar sensitivity and specificity for detecting precancerous lesions to samples collected by a physician or nurse [5,6,7] and is considered a safe and acceptable screening method by users [8,9,10,11,12]. This approach reduces the discomfort and pain that can be associated with pelvic examination, is considered less invasive, allows women more autonomy and privacy, and might facilitate screening participation [8,13,14]. In the context of primary screening, a negative HPV result indicates a very low risk of developing CC within the next decade and a positive result can generally be safely managed by adequately trained health care providers [15].

Self-HPV provides an opportunity to increase screening coverage to 70% of eligible women as recommended by the WHO. However, there are still some uncertainties about the introduction of this method for all women engaged in routine screening campaigns. The main uncertainties reported by participants are the quality of the sample, lack of confidence that they will perform the test correctly, and the reliability of the results [16].

The above results were mostly focused only on the acceptability of self-sampling and were not contextualised to sub-Saharan African countries. For these reasons, our study aimed to evaluate sociodemographic factors associated with preference for conventional HPV clinician-sampling compared with Self-HPV, in order to propose effective screening strategies that are aligned with the WHO’s recommendations. Currently the Cameroonian health system supports and follows the WHO’s recommendations for cervical cancer screening. There is no effective national screening programme, but early opportunistic screening for cervical cancer by visual inspection with acetic acid and Lugol’s iodine (VIA/VILI) is promoted and sporadic campaigns are organized [17].

## 2. Materials and Methods

### 2.1. Setting and Study Design

This study was embedded in the research project “Comprehensive Cervical Cancer Prevention and Better Women Health in Medium and Low-Resource Setting” launched in 2018 [15] by the University of Dschang, the Dschang District Hospital, the University of Yaoundé in Cameroon, and the University Hospital of Geneva, Switzerland [18].

### 2.2. Study Procedures

The research project was based on a “3T-Approach” (for “same-day test, triage and treatment”). After a one-hour health education group session, midwives individually provided detailed information about the study and collected written informed consent. A baseline survey including sociodemographic characteristics and medical history was also conducted. Self-HPV was performed by participants using flocked swabs (FLOQSwabs^®^ Self Collection; Copan, CA, USA). Before performing the self-test, women received instructions and a support guide that provided detailed visual information about the procedure. They were instructed to wash their hands before the procedure; they then performed the test in a dedicated area within the screening room with a midwife available if needed. Instructions directed the women to hold the plastic head of the swab and insert the cotton tip into the vagina until it met resistance. Specimens were analysed by a point-of-care HPV test (GeneXpert^®^) using the Xpert HPV Assay (Cepheid, Sunnyvale, CA, USA), and results were available after one hour.

Women screened positive underwent visual inspection with acetic acid and Lugol’s iodine (VIA/VILI) to detect whether pre-cancerous or cancerous lesions were visible. If the VIA/VILI result was positive, women were treated by thermal ablation or loop electrosurgical excision of the transformation zone (LEETZ) according to predefined eligibility criteria. Quality control was ensured by histological sampling (biopsies and endocervical curettage), which was considered the gold standard.

### 2.3. Acceptability of and Satisfaction with the HPV Self-Sampling Procedure

After the self-sampling procedure and before receiving the result, women filled in a second survey in their native language. With the assistance of a midwife, the participants reported how they felt during the test, their satisfaction with the procedure, their levels of anxiety and discomfort, their willingness to take the test again, whether they would recommend the test, and where they thought the test should be conducted (at home vs. at a health care centre). Pain experienced during the self-sampling procedure was scored according to the Wong-Baker FACES^®^ scale [19]. This validated scale consists of six different faces with a spectrum of pain intensity from 0 (no hurt) to 10 (hurts worst). We then formed two subgroups: medium pain (score ≤ 4) and strong pain (score > 4). A four-point Likert-type scale was used to evaluate the women’s anxiety, discomfort, embarrassment, and level of confidence with scores ranging between 1 (not at all) to 4 (very). Study data were collected with paper Case Report Forms (p-CRF) by trained midwives and later transcribed into an electronic database (SecuTrial^®^).

### 2.4. Ethical Considerations

The protocol obtained approval from the Cantonal Ethics Board of Geneva, Switzerland (Commission cantonale d’éthique de la recherche, N °2017-01110) and the National Ethics Committee for Research on Human Health, Cameroun (Comité national d’éthique de la recherche pour la santé humaine, CNERSH, N °2018/07/1083/CE/CNERSH/SP). The study protocol of the overarching 3T Study was registered under ClinicalTrials.gov (number NCT03757299).

### 2.5. Statistical Analysis

Quantitative variables were expressed as means and standard deviations, and qualitative variables were expressed as percentages unless otherwise stated. Descriptive analyses were conducted to compare baseline sociodemographic and clinical characteristics according to the preference for clinician-sampled versus Self-HPV. Categorical variables were analysed by Pearson’s chi-square or Fisher’s test when appropriate. In addition, we used univariable and multivariable logistic regression models to identify sociodemographic factors associated with preference for HPV self-sampling. Education, employment status, and parity were used in the multivariable logistic regression model, and were selected based on their *p*-value in the univariable models. We used a two-sided level of significance of 0.05. The analyses were conducted using the software package STATA^®^ 16 (Stata, College Station, TX, USA).

## 3. Results

### 3.1. Epidemiological Characteristics

A total of 2201 women were enrolled in the 3T-Approach between September 2018 and January 2021. Median age was 40.6 (IQR 35–45) years. Among participants, 406 (18.4%) were HPV-positive, and 176 (8%) had been previously screened (Table 1). Most (76.9%) preferred HPV self-sampling or had no preference, and 508 (23.1%) preferred clinician-sampling. Demographic characteristics of the participants are shown in Table 1 and stratified by procedure preference (self-sampled or neutral versus (vs.) clinician-sampled). There was a higher proportion of women with a tertiary education level in the clinician-sampling preference group (29.5%) than in the Self-HPV preference or neutral group (14.4%, *p* < 0.001). Similarly, the proportion of women employed with higher grade professional or managerial occupations was higher in the clinician-sampling preference group (5.5% vs. 2.6%, *p* = 0.005). The Self-HPV preference group had a lower proportion of nulligravida than the clinician-sampling preference group (1.9% vs. 3.0%; *p* = 0.001) and a higher proportion of women with more than five pregnancies (56.6% vs. 47.4% *p* < 0.001). There was a higher proportion of women with more than five children in the self-sampling preference group than in the clinician-sampling preference group (35.8% vs. 26.1%; *p* < 0.001). No difference was found concerning previous HPV sampling by clinicians between the two groups.

### 3.2. Acceptability of the HPV Self-Sampling Procedure by Sampling Preference

**Figure 1:**
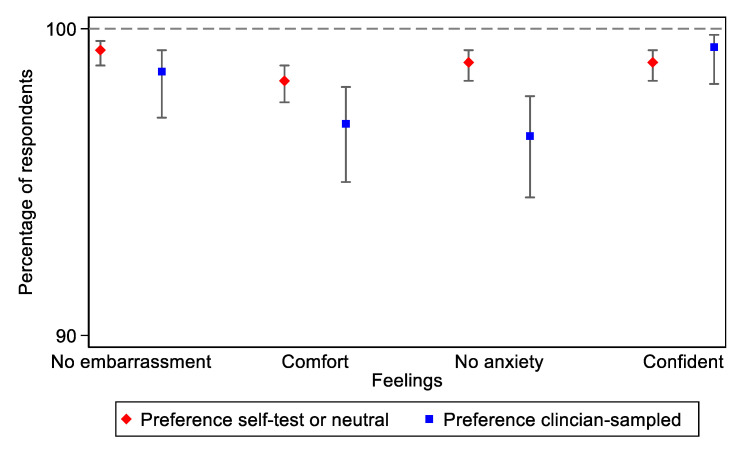
Comparison of women’s experiences with Self-HPV (preference for self-test or neutral vs. clinician-sampled).

Most women (98.3% in self- and 96.9% in clinician-sampling; *p* = 0.053) reported to be comfortable or very comfortable and confident or very confident (98.9% vs. 99.4%; *p* = 0.341) during the self-sampling procedure. No significant differences were reported. The same observation was made among women who expressed no or low embarrassment (99.3% vs. 98.6%; *p* = 0.107). However, among women who described feeling anxious (1.1% vs. 3.5%; *p* < 0.001), we found that women who preferred clinician-sampling were significantly more anxious (Figure 1). The source of anxiety reported by most participants (86.0% vs. 77.3%) was fear of a positive result, followed by fear of the self-sampling procedure (6.8%) in the clinician-sampled group, and fear of doing the self-test for the first time (4.3%) in the self-test preference group.

Few participants reported difficulties in performing the Self-HPV (1.2% vs. 1.6%; *p* = 0.488, in the self-sampled or neutral and clinician-sampled preference groups, respectively). Fewer than 0.05% (1/2201) women reported pain (score >4/10) during Self-HPV across the two groups. Most women in both preference groups were willing to repeat the self-test as a screening test (99.8% vs. 99.2%; *p* = 0.035), and they agreed to perform it at home (98.4% vs. 94.9%; *p* < 0.001). Only one woman in the self-sampled preference group and one in the clinician-sampled preference group reported that they would not recommend the procedure to their relatives. (Table 2).

Univariate logistic regression showed an association between sociodemographic variables and preference for the clinician-sampled procedure. In particular, education level was significantly higher among women preferring clinician-sampling. Women having attended tertiary education were more than three times more likely to prefer clinician-sampling than women with primary education or no formal education (OR, 3.13; 95% CI 2.34–4.19). Women having attended secondary education were 1.42 times more likely (95% CI 1.10–1.82) to prefer clinician-sampling. These findings show a gradient in the association between level of education and preference for clinician-sampling. Compared with unpaid workers, women employed with higher grade professional or managerial occupations were more likely to prefer clinician-sampling (OR, 2.08; 95% CI 1.25–3.48). However, no difference in preference was observed among women employed with lower grade or intermediate occupation or self-employed women compared with unpaid workers (OR, 0.96; 95% CI 0.76–1.21). Parity was also associated with sampling preference, with women having more than five children being less likely to prefer clinician-sampling (OR, 0.52; 95% CI 0.32–0.85) compared with nulliparous women.

In the multivariable logistic regression model, parity and employment status were no longer significantly associated with sampling preference. However, the gradient between level of education and preference for clinician-sampling remained, with women with a secondary or tertiary level of education showing a significant preference to undergo clinician-sampling (OR, 1.35; 95% CI 1.05–1.75, and OR, 2.79; 95% CI 2.03–3.82, respectively) (Table 3).

### 3.3. Perceptions Regarding the Sampling Procedure

Most reasons for preferring self-sampling over clinician-sampling were its ease of use (75.2%) and the possibility for increased privacy (16.3%). The top reasons women gave for preferring clinician-sampling over self-sampling were feeling comfortable because of the clinician’s greater experience (76.1%) and the higher reliability of the results (19.5%). The possibility to undergo a complete gynaecological exam (2.5%) was a less common reason reported by women, as was the belief that sampling is the role of caregivers (0.8%) (Table 4). Most participants preferred performing self-sampling in a medical centre (96.6%) over home-based screening, because they were afraid of performing the self-test inappropriately (36%) or contaminating it (12%) if performed at home.

## 4. Discussion

HPV self-sampling may be an effective approach to cervical cancer screening programs, although there are still challenges affecting its implementation. Because low-resource settings are progressively introducing both HPV-based primary screening and Self-HPV sampling [20,21], we sought to explore factors associated with women’s preference for sampling performed by health care providers in this context. Our study was conducted in a population where most participants had never undergone screening before (92.0%). Our findings show that most women consider Self-HPV easy to use (97.9%), as previously reported in the literature [22]. More than 99.5% of the women reported that they would agree to do it again, and 97.6% would agree to perform it at home.

Some women (23.1%) preferred sampling performed by clinicians, as was previously reported in other studies [8,23,24,25]. In Cameroon, an earlier study explored the perceptions and preferences regarding self- versus physician-sampling in a population of women living with HIV and observed that most preferred clinician-sampling [26]. This was found in another study conducted in Britain among Muslim women interviewed in the context of cervical cancer screening [24]. The reasons for preferring clinician-sampling in these studies were concerns about not doing the test correctly and the belief that health providers are more expert (95.6%), which is consistent with our findings. In the literature, women also reported having a low level of self-confidence performing self-sampling properly and providing a good quality sample for analysis [8,16,27,28,29].

Sociodemographic factors associated with women’s preferences show that well-educated women prefer clinician-sampling. Similar findings were reported in Malaysia, where women with a high education level presented a low level of confidence in self-testing [22]. In China, authors investigated the preference for self-testing for streptococcus B infection instead of clinician-sampling, and their findings showed a similar tendency, that women with the highest education level were more likely to prefer clinician-collection [30]. The finding that women with a higher education prefer clinician-sampling may be explained by their increased questioning of their ability to perform the test themselves adequately [22]. Being able to afford going to a hospital to seek care may also influence the preference to have a clinician conduct cervical cancer screening. However, in a different study on vaginal HPV self-sampling, the authors found no association between level of education and preference for procedure type [31]. Cultural beliefs may also influence patients’ perception of the self-sampling approach and the idea that health care provider attendance is needed during the procedure [22,29,32]. Qualitative studies could be useful to further explore reasons for clinician- versus self-sampling preferences.

Educational interventions were highlighted as an effective way to improve women’s self-confidence and clarify misconceptions [29,33]. Instruction about the validity of the test and how to collect the sample should be simple and should account for the women’s cultural and social characteristics [13]. Women who have never used tampons or do not feel comfortable about touching their genital areas perceive this practice as taboo and appear less confident about performing a self-testing procedure [13,16]. Allowing them to observe and manipulate a swab during the educational intervention is one way to reduce their worries [32]. The presence of a healthcare provider during Self-HPV has been reported by some authors to have a positive effect on women. Support is therefore a way of increasing women’s confidence and comfort [8,28], but the option to choose clinical-sampled could be a solution for women who are unwilling to undergo Self-HPV, as was proposed in Australia [34]. In our program, an hour of educational and counselling intervention provided to women before performing the Self-HPV probably allows sufficient time to respond to the women’s specific fears. Health care providers need to fully understand the Self-HPV screening procedure and should be able to give culturally appropriate messages to participating women [8]. They also need to be prepared to answer any concerns that women may have regarding Self-HPV. This requires rigorous theoretical and practical training of health care providers.

Community health workers and peer-to-peer education also contribute to increasing women’s awareness and confidence in Self-HPV by providing them with adequate information [32]. The Cameroonian health system already provides an important value to the community approach to health promotion in the population, as is also the case in other African countries [32]. Political endorsements and health system support are key in this screening process and in health care training [35]. Recommendations on screening and management strategies for cervical cancer elimination from previous research should be applied at a state level, as is the case in Uganda, Rwanda, and Kenya, where these recommendations have been introduced into national guidelines [20,21,36].

The strength of this study lies in the large sample size of women recruited in real-world screening conditions. In addition to the Self-HPV acceptability analysis, we assessed women’s preferences regarding the screening procedure. Identifying associations between sociodemographic characteristics and screening preference contributes to providing adapted instructions to women and improving their experiences, thus increasing screening coverage.

There are two main limitations to this study. First, for illiterate women or those who were not comfortable answering independently, the questionnaires were completed with the help of midwives. This could have influenced the women’s answers if any women were not comfortable discussing their feelings about intimacy-related topics in the presence of midwives. Second, the women recruited in our study and who took part in our screening program come from a constrained health area and have a higher level of education on average than the general female population in Cameroon [37]. Therefore, our results may not be generalizable to all women in Cameroon.

## 5. Conclusions

Self-HPV is highly accepted by women and is an effective method of detecting pre-cancerous lesions. Our findings support a self-sampling approach as primary screening method. However, implementation of a community-based Self-HPV approach needs to be appropriate to the local context. Health systems should assist participants with the screening process to increase self-confidence about the accuracy of self-sampling. Given that a minority of participants prefer clinician-based sampling, an approach where women can choose the sampling method should be considered.

## Figures and Tables

**Table 1:** Baseline sociodemographic and clinical characteristics according to preference for clinician-sampled or self-sampled HPV test.

Variable	Preference for Self-Sampled or Neutral N (%)	Preference for Clinician-Sampled N (%)	*p*-Value
Participants recruited (n = 2201)	1693 (76.9)	508 (23.1)	
HPV testing results (n = 2201)			0.179
Negative	1391(82.2)	404 (79.5)	
Positive	302 (17.8)	104 (20.5)	
Age (y), mean ± SD	39.9 (5.9)	39.1 (6.0)	0.001
Marital status (n = 2198)			0.840
Single/divorced/widowed	257 (15.2)	75 (14.8)	
Married/in relationship	1435 (84.8)	431 (85.2)	
Education (n = 2195)			<0.001
Unschooled/primary education	541 (32.0)	106 (21.0)	
Secondary education	905 (53.6)	251 (49.6)	
Tertiary education	243 (14.4)	149 (29.5)	
Employment status (n = 2198)			0.006
Unpaid worker *	422 (25.0)	126 (24.9)	
Lower grade or intermediate occupation **/self-employed	1224 (72.4)	352 (69.6)	
Higher grade occupation ***	45 (2.6)	28 (5.5)	
Age at first delivery (y), mean ± SD	20.7 (5.1)	21.4 (6.2)	0.006
Age at first intercourse (y) mean ± SD	17.9 (2.7)	18.0 (3.0)	0.354
Pregnancy (n = 2198)			0.001
Nulligravida	32 (1.9)	15 (3)	
1–5	702 (41.5)	251 (49.6)	
>5	958 (56.6)	240 (47.4)	
Parity (n = 2198)			<0.001
Nulliparous	62 (3.7)	26 (5.1)	
1–5	1024 (60.5)	348 (68.8)	
>5	606 (35.8)	132 (26.1)	
Previous HPV screening (n = 2197)			0.405
No	1560 (92.3)	461 (91.1)	
Yes	131 (7.7)	45 (8.9)	
HIV status (self-reported) (n = 2156)			0.535
Negative	1601 (96.4)	480 (94.5)	
Positive	60 (3.6)	15 (5.5)	

Note: N, number; HPV, human papillomavirus; y, years; SD, standard deviation; HIV, Human Immunodeficiency virus, * Unpaid worker (ex: student, housewife), ** Employee with lower grade or intermediate occupation (ex: nurse, technician, and teacher), *** Employee with higher grade professional administrative or managerial occupations (ex: doctor, manager, school director).

**Table 2:** Comparison of feelings about HPV self-sampling between preference groups.

Feelings about Self-Sampling	Preference for Self-Sampled or Neutral N (%)	Preference for Clinician-Sampled N (%)	*p*-Value *
Agree to repeat self-sampling	1687 (99.8)	504 (99.2)	0.035
Agree to perform self-sampling at home	1666 (98.4)	481 (94.9)	<0.001
Difficult to perform self-sampling	20 (1.2)	8 (1.6)	0.488
Would recommend self-sampling			0.573
Yes	1687 (99.9)	507 (99.8)	
No	1 (0.1)	1 (0.2)	

* Fisher’s exact test used to account for low cell counts.

**Table 3:** Association of sociodemographic factors with preference for clinician-sampled.

Sociodemographic Variables	Clinician-Sampled			
	Unadjusted		Adjusted	
	OR * (95% IC)	*p*-Value	OR (95% IC)	*p*-Value
Education				
Unschooled/primary education	Ref		Ref	
Secondary education	1.42 (1.10–1.82)	0.007	1.35 (1.05–1.75)	0.019
Tertiary education	3.13 (2.34–4.19)	<0.001	2.79 (2.03–3.82)	<0.001
Employment status				
Unpaid worker *	Ref		Ref	
Lower grade or intermediate occupation **/self-employed	0.96 (0.76–1.21)	0.751	0.91 (0.72–1.15)	0.435
Higher grade occupation ***	2.08 (1.25–3.48)	0.005	1.35 (0.79–2.30)	0.272
Parity				
Nulliparous	Ref		Ref	
1–5	0.81 (0.50–1.30)	0.384	1.06 (0.65–1.73)	0.824
>5	0.52 (0.32–0.85)	0.010	0.84 (0.50–1.43)	0.523
Pregnancy				
Nulligravida	Ref			
1–5	0.76 (0.41–1.43)	0.400		
>5	0.53 (0.28–1.00)	0.051		
Marital status				
Married/in relationship	Ref			
Single/divorced/widowed	0.97 (0.74–1.28)	0.840		

Note: OR, odds ratio; CI, confidence interval. * housewife or student; ** Lower grade professional, administrative and managerial occupations and higher grade technician and supervisory occupations, *** Large employers, higher grade professional, administrative and managerial occupations.

**Table 4:** Reasons for preferring HPV self-sampling or clinician-sampling *.

Reasons for preferring self-sampling (n = 479)	N	(%)
Easy and rapid	360	75.2
Affords privacy	78	16.3
Autonomous	11	2.3
Fear of gynaecological examinations	11	2.3
Reliability of results	7	1.5
Self-confidence	6	1.3
New learning experience	6	1.3
Reasons for preferring clinician-sampling (n = 486)		
Expertise of clinician	370	76.1
Reliability of results	95	19.5
Possibility of inspecting the cervix	12	2.5
Caregiver’s role	4	0.8
Difficulty of performing the test	3	0.6
Ease of procedure	2	0.4

* Multiple responses allowed.

## Data Availability

The datasets analysed during this study are available from the corresponding author upon request.
